# Complete Mitochondrial Genome Analysis Reveals Genetic Diversity in the Narrow–Ridged Finless Porpoise (*Neophocaena asiaeorientalis*) Across East Asian Waters

**DOI:** 10.1002/ece3.71229

**Published:** 2025-04-10

**Authors:** Sunmin Kim, Youngran Lee, Dong‐Yeop Lee, Heesu Lee, Seon‐Mi Lee, Nari Kim, Seongjun Choe, Dong‐Hun Lee

**Affiliations:** ^1^ Department of Parasitology, School of Medicine and Parasite Research Center Chungbuk National University Cheongju Republic of Korea; ^2^ Plan Ocean Seoul Republic of Korea; ^3^ Wildlife Health Laboratory, College of Veterinary Medicine Konkuk University Seoul Republic of Korea; ^4^ National Institute of Wildlife Disease Control and Prevention Gwangju Republic of Korea; ^5^ College of Veterinary Medicine Chungbuk National University Cheongju Republic of Korea; ^6^ Konkuk University Zoonotic Disease Research Center Konkuk University Seoul Republic of Korea

**Keywords:** complete mitogenome, conservation genetics, narrow‐ridged finless porpoise, *Neophocaena asiaeorientalis*, Sample location: Korea

## Abstract

The narrow‐ridged finless porpoise ( *Neophocaena asiaeorientalis* Pilleri & Gihr 1972) is one of the most endangered cetacean species inhabiting East Asian waters. Complete mitogenome analysis offers accurate phylogenetic insights; however, complete mitogenome sequences for the narrow‐ridged finless porpoise have so far been restricted to specific regions, mainly in China, and no sequences are available from Korean or Japanese populations. To address this gap, in this study, we developed a multiplex PCR primer panel to sequence the complete mitochondrial genome of the porpoise and sequenced 23 individuals of *N. a. sunameri*, a subspecies of *N. asiaeorientalis* , from Korean waters using next‐generation sequencing. Phylogenetic analyses based on maximum likelihood and Bayesian inference revealed three major, well‐supported monophyletic clades within the species. Two sequences of the Yangtze finless porpoise (*N. a. asiaeorientalis*), another subspecies, displayed significantly higher genetic divergence compared to *N. a. sunameri* sequences. The 23 mitochondrial genome sequences exhibited a nucleotide diversity of 0.142% and a haplotype diversity of 99.6%, with 22 unique haplotypes identified. These findings contribute to our understanding of the evolutionary history and genetic diversity of the species, providing valuable insights for future conservation efforts and further genetic research.

## Graphical abstract

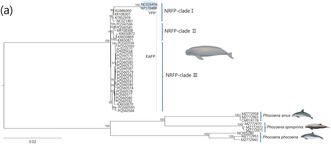

## 1 Introduction

The genus *Neophocaena* (Palmer, 1899) includes small phocoenid cetaceans found in Asian waters (Wilson and Reeder 2005). These cetaceans, commonly referred to as “finless porpoises” due to their lack of a dorsal fin, are currently represented by two recognized species: *N. phocaenoides* (Cuvier, 1829) and *N. asiaeorientalis* (Pilleri and Gihr 1972). These species, distinguished by their dorsal ridges, coexist in the waters around the Taiwan Strait but have distinct distributions (Wang et al. 2010; Amano 2018). *Neophocaena asiaeorientalis* is further divided into two subspecies: *N. a. sunameri* (East Asian finless porpoise, Pilleri and Gihr 1975), which inhabits the shallow coastal waters of East Asia (Amano 2018), and *N. a. asiaeorientalis* (Yangtze finless porpoise, Rudolph and Smeenk 2009), found in the Yangtze River and nearby lakes in China (Rudolph and Smeenk 2009). Recent conservation assessments have raised concerns about the risk to these populations, with some studies proposing that *N. a. asiaeorientalis* be recognized as a distinct species due to its genetic isolation (Zhou et al. 2018).

The mitochondrial genome (mitogenome or mtDNA) evolves more rapidly than the nuclear genome (nuDNA), making it a valuable resource for studying genetic divergence (Nei 1987). Due to its low recombination rate and high sensitivity to recent speciation events, the mitogenome more effectively captures hierarchical relationships among closely related taxa and within species than nuDNA (DeSalle et al. 2017; Sodhi et al. 2022). While the mitochondrial cytochrome c oxidase 1 (*cox1*) gene is commonly used for many animal groups (Hebert et al. 2003), cetacean studies have primarily relied on the mitochondrial DNA control region (CR, also known as the D‐loop) and cytochrome b (*cyt b*) gene for genetic analyses (Árnason et al. 1993; Milinkovitch et al. 1994; Árnason and Gullberg 1996; Baker and Dalebout 2009). Research on the genus *Neophocaena* has also employed these markers (Yang et al. 2002; Wang et al. 2008; Li et al. 2011). Although recent studies have explored the genus' phylogeny, genetic diversity, phylogeography, and population demography with larger sample sizes (Lin et al. 2014, 2017; Jia et al. 2014; Lee et al. 2019), contributing to a better understanding of their evolutionary history, a common limitation has been that the sequence lengths used were insufficient to fully assess genetic diversity and differentiation. Furthermore, recent evidence suggests that the mtDNA CR and *cyt b* genes may not provide adequate resolution for accurately resolving phylogenetic relationships among closely related taxa, especially in cases of introgression (Chehida et al. 2020).

To address these limitations, complete mitogenome analysis, which generally offers more accurate phylogenetic insights, has been recognized as the most reliable approach for achieving a comprehensive understanding of a species' evolutionary history and genetic diversity. It is crucial to understand these aspects to establish effective conservation strategies (Dufresnes et al. 2013; Moritz and Potter 2013; Chehida et al. 2020), particularly for endangered species inhabiting limited regions such as the narrow‐ridged finless porpoise. However, complete mitogenome sequences for the narrow‐ridged finless porpoise have so far been restricted to specific regions, mainly in China, and no sequences are available from Korean or Japanese populations. As noted by Lee et al. (2019), the narrow‐ridged finless porpoise population in Korean waters exhibits the highest genetic diversity and lowest genetic differentiation among East Asian populations, underscoring its critical role in species conservation. Therefore, the objectives of this study were: (1) to obtain complete mitochondrial DNA sequences from the Korean population of narrow‐ridged finless porpoises, (2) to analyze the phylogenetic relationships and elucidate the species' evolutionary history, and (3) to assess the genetic diversity of the Korean population.

## 2 Materials and Methods

A total of 23 narrow‐ridged finless porpoise carcasses, either stranded or bycaught off the Korean coast, were subjected to necropsy between 2021 and 2024. Permission for the collection of these carcasses was granted by the appropriate local and regional authorities. Specifically, for each carcass, a Cetacean Disposal Certificate and a Cetacean Transfer Agreement were issued by the jurisdictional maritime police, and permission for the storage of protected marine species was obtained from the local authorities before conducting necropsies. This ensured that all procedures complied with the relevant legal and environmental mandates.

**FIGURE 1:**
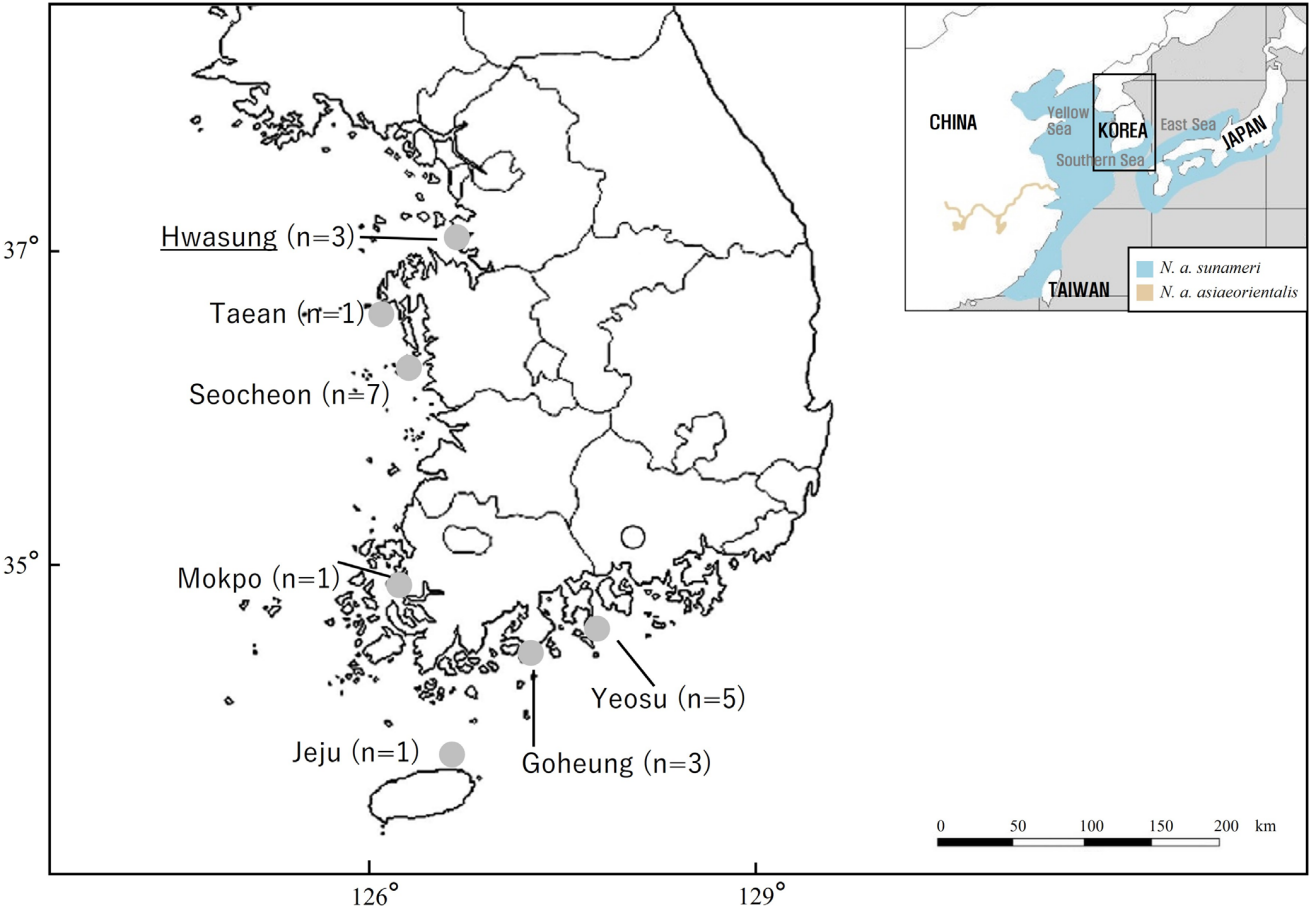
Collection locations of East Asian finless porpoise carcasses with corresponding specimen numbers; the origins of two specimens are unknown.

Of these, nine individuals were collected from the Southern sea of Korea, including Jeju Island, while the remaining 12 were found in the Korean Yellow Sea. Two carcasses were of unknown origin. Interestingly, narrow‐ridged finless porpoises are not known to inhabit the waters near Jeju Island, leaving the origin of one of the carcasses unclear. During necropsy, muscle and internal organ tissues were collected and preserved in 70% ethanol for molecular analysis. Table 1 provides information about the specimens, including specimen ID, collection site, and sea area, while Figure 1 illustrates the collection locations along the Korean coast.

**TABLE 1:** Information on the East Asian finless porpoise specimens used in this study.

ID	Collection date	Collection site	Collection sea area	GenBank accession number
NA20‐1128A	2020.11.28	Jeju Island (33°33.48, 126°44.69)	SS	PQ560572
NA21YS‐0328	2021.03.28	Seocheon (36°02.50, 126°52.50)	KYS	PQ560573
NA21YS‐1115	2021.11.15	Seocheon (36°09.11, 126°28.23)	KYS	PQ560574
NA21YS‐1118	2021.11.18	Seocheon (36°10.14, 126°20.57)	KYS	PQ560575
NA21YS‐1119	2021.11.19	Seocheon (36°07.07, 126°23.24)	KYS	PQ560576
NA21YS‐1123	2021.11.23	Taean (exact point unknown)	KYS	PQ560577
NA22YS‐1008	2022.10.08	Seocheon (36°08.92, 126°17.81)	KYS	PQ560578
NA22YS‐1012	2022.10.12	Yeosu (34°35.35, 127°42.58)	SS	PQ560579
NA22YS‐1120	2022.11.20	Goheung (34°46.50, 127°08.55)	SS	PQ560580
NA23YS‐0221	2023.02.21	Yeosu (34°62.92, 127°71.92)	SS	PQ560581
NA23YS‐0325	2023.03.25	Hwaseong (37°16.55, 126°61.70)	KYS	PQ560582
NA23YS‐0327	2023.03.27	Hwaseong (37°12.08, 126°68.46)	KYS	PQ560583
NA23YS‐0407	2023.04.07	Goheung (34°55.61, 127°11.50)	SSSS	PQ560584
NA23YS‐0408	2023.04.08	Hwaseong (37°14.88, 126°68.61)	KYS	PQ560585
NA23YS‐0521	2023.05.21	Goheung (34°62.94, 127°49.59)	SS	PQ560586
NA23YS‐0529	2023.05.29	Yeosu (34°44.44, 127°39.06)	SS	PQ560587
NA23YS‐0615	2023.06.15	Mokpo (35°07.44, 126°36.43)	KYS	PQ560588
NA23YS‐1022	2023.10.22	Yeosu (34°81.88, 127°76.72)	SS	PQ560589
NA23YS‐1101A	2023.11.01	Seocheon (36°06.15, 126°15.83)	KYS	PQ560590
NA23YS‐1101B	2023.11.01	Seocheon (36°06.15, 126°15.83)	KYS	PQ560591
NA24YS‐0227	2024.02.27	Yeosu (34°53.17, 127°76.94)	SS	PQ560592
NA23YS‐unknown1	Unknown	Unknown	Unknown	PQ560593
NA23YS‐ unknown2				PQ560594

* SS= Southern Sea of Korea, KYS= Korean Yellow Sea

Genomic DNA was extracted from the tissue samples using the DNeasy Blood & Tissue Kit (Qiagen, Valencia, CA), following the manufacturer's protocol. The complete mitogenome was captured using the multiplex tiling PCR method. Initially, available mtDNA genomes of narrow‐ridged finless porpoises were downloaded from the National Center for Biotechnology Information (NCBI) database and aligned to generate a consensus sequence. Primers were then designed using the PrimalScheme software version 3.0.2 (https://primalscheme.com/) to amplify the entire genome (Table 2). DNA samples were subjected to multiplex PCR using the TaKaRa Ex Taq DNA polymerase kit (Takara Bio Inc., Shiga, Japan), following the manufacturer's instructions. The PCR mixture consisted of 5 μL of 10X Ex Taq buffer, 4 μL of dNTP mix, 0.6 μM of each primer, 0.5 μL of Ex Taq polymerase, 2 μL of template DNA, and 36.5 μL of nuclease‐free water. PCR amplification conditions were as follows: initial denaturation at 94°C for 5 min, followed by 40 cycles of 94°C for 1 min, 57°C for 30 s, and 72°C for 1 min 30 s. The PCR products, showing approximately 1.5‐kbp amplicons, were visualized on 1% agarose gels. PCR products were pooled, and approximately 100,000 next‐generation sequencing (NGS) reads of 150 bp per sample were generated using the Illumina MiniSeq sequencing system (Illumina, San Diego, CA).

**TABLE 2:** Primer set used in porpoise mtDNA multiplex PCR.

Primer set	Primer sequence (5′–3′)	Product length (bp)	Position (nt)
1F	GCCCCATCAACACAAAGGTTTG	2098	59–2098
1R	CCTCGTGTGGCCATTCATACAA		
2F	CAGGCGTGCACTAAGGAAAGAT	2036	1883–3918
2R	ACCAACATTTTCGGGGTATGGG		
3F	ATTCCTCCACAAGCCTAGACCT	1987	3704–5690
3R	AAAGAGGGAGGAAGCAGTCAGA		
4F	AACCTGGCTCACTTATCGGAGA	2068	5492–7559
4R	CTAGAGAGGGGACGGCTCAT		
5F	ACAACCCCTCCTTGACCGTA	2027	7326–9352
5R	GCCAAAGTGGTGGTTTGATGTG		
5F’	CTCTTCGCGCTACTCCAAC	1366	7868–9233
5R’	ACCCGTAGACTCCGTCTG		
6F	TGGCCTCTATTTCACCCTCCTT	1946	9157–11,102
6R	GACGAGTGCTATGTGGCTGATT		
7F	GAAGCCCCTATTGCAGGTTCAA	2040	10,875–12,914
7R	AGTGATGAGGGCGGTTGTAGTA		
8F	ACCTCTAGCCAACTTGGTCTGA	2048	12,705–14,752
8R	TGGGAGAATAAAGTGGAAGGCG		
9F	ACACTACACACCAGACACCTCT	1944	14,353–16,296
9R	TAGGGACCAAGCAGTCTAAGGG		
10F	GTCTGCTTTAATATTCACC	813	16,253–681
10R	ATAGACTGAAGTAGCAAGG		

Raw reads were processed to remove adapters and low‐quality bases using BBDuk version 38.84, with a minimum quality threshold of 20 (Bushnell 2014). Mitochondrial genome assemblies were conducted both de novo and using reference‐based methods. For reference‐based assembly, trimmed reads were mapped to the *Neophocaena phocaenoides* isolate WH‐6‐NP mitochondrial genome (GenBank accession number: KX650870) using Minimap2 version 2.24 with default settings (Li 2018). Assembly results were visualized using Geneious Prime version 2022.2.2 (Kearse et al. 2012). De novo assembly was performed using the SPAdes assembler version 3.15.5 (Bankevich et al. 2012). Final consensus genome sequences were generated by integrating results from both methods. Regions with insufficient coverage in the NGS data were supplemented using Sanger sequencing with additional primers (Table 2).

To assess the phylogenetic position of narrow‐ridged finless porpoises, complete mitogenome sequences from 11 individuals of the genus *Neophocaena* and nine individuals from three species of the genus *Phocoena* were retrieved from the GenBank database (Table 3). Sequences with high ambiguities were excluded. Sequence alignment was conducted using MAFFT version 7.490 (Katoh and Standley 2013) and MEGA X version 10.1.7 with ClustalW (Larkin et al. 2007). Phylogenetic trees were constructed using maximum likelihood (ML) and Bayesian inference (BI) methods. For ML analysis, RAxML version 8.2.13 was used with the GTR + G substitution model and 1000 bootstrap replicates (Stamatakis 2014). For BI analysis, MrBayes version 3.2.6 was applied using the HKY + I + G model, running for 10 million generations (Ronquist et al. 2012). The best‐fit evolutionary substitution models were selected using jModelTest version 2.1.10 (Darriba et al. 2012), based on the Bayesian Information Criterion (BIC). Phylogenetic trees were visualized using FigTree version 1.4.4 (Rambaut 2012).

**TABLE 3:** List of sequences used in the phylogenetic analyses along with GenBank accession numbers, collection sites, references, and estimated species inferred from the phylogeny results, including sequences generated in this study.

	Registered species	Genebank accession number	Collection site	Reference	Inferred species
Phocoenidae					
*Neophocaena*	*N. asiaeorientalis. asiaeorientalis*	KP170488	Yantze river	Liu et al. (2016)	*N. a. asiaeorientalis*
	*N. asiaeorientalis. sunameri*	KR108307	Yantze River Estuary	Unpublished	*N. a. sunameri*
	*N. asiaeorientalis. asiaeorientalis*	KR108308	Northern Yellow Sea	Unpublished	*N. a. sunameri*
	*N. asiaeorientalis. sunameri*	KT852939	East China Sea	Cheng et al. (2017)	*N. a. sunameri*
	*N. asiaeorientalis. asiaeorientalis*	KU886000	Yantze river	Unpublished	*N. a. sunameri*
	*N. asiaeorientalis. sunameri*	KX650869	Unknown	Unpublished	*N. a. sunameri*
	*N. phocaenoides*	KX650870			*N. a. sunameri*
	*N. asiaeorientalis. sunameri*	KX650871			*N. a. sunameri*
	*N. asiaeorientalis. sunameri*	KX650872			*N. a. sunameri*
	*N. phocaenoides*	NC_021461 (=KC777291)	Zhejiang Province, East China Sea, Zhoushan fishing grounds	Unpublished	*N. a. sunameri*
	*N. asiaeorientalis*	NC_026456	Unknown	Liu et al. (2016)	*N. a. asiaeorientalis*
	*N. asiaeorientalis. sunameri*	PQ560572–PQ560594	Korean seas	This study	*N. a. sunameri*
*Phocoena* (outgroup)	*P. phocoena*	NC_005280		Arnason et al. (2004)	
		MZ772943		Morin et al. (2023)	
		MZ772953			
	*P. sinus*	CM018178		Morin et al. (2021)	
		MZ772958		Morin et al. (2023)	
		MZ772965			
	*P. spinipinnis*	MZ772970			
		MZ772971			
		MZ772972			

In addition to complete mitogenome analysis, partial sequence analyses were conducted using two widely applied cetacean markers: the mtCR and *cyt b* genes, along with the *cox1* marker, commonly used in other taxonomic groups. The partial sequences used for these analyses are also listed in Table 3. The ML approach was used with the same methodology as described above. Genetic diversity and polymorphic sites in the Korean population of narrow‐ridged finless porpoises were evaluated using DnaSP version 6.12.03 (Librado and Rozas 2009), with nucleotide and haplotype diversity estimates following Nei (1987).

## 3 Results

### 3.1 Phylogenetic Analyses

**FIGURE 2:**
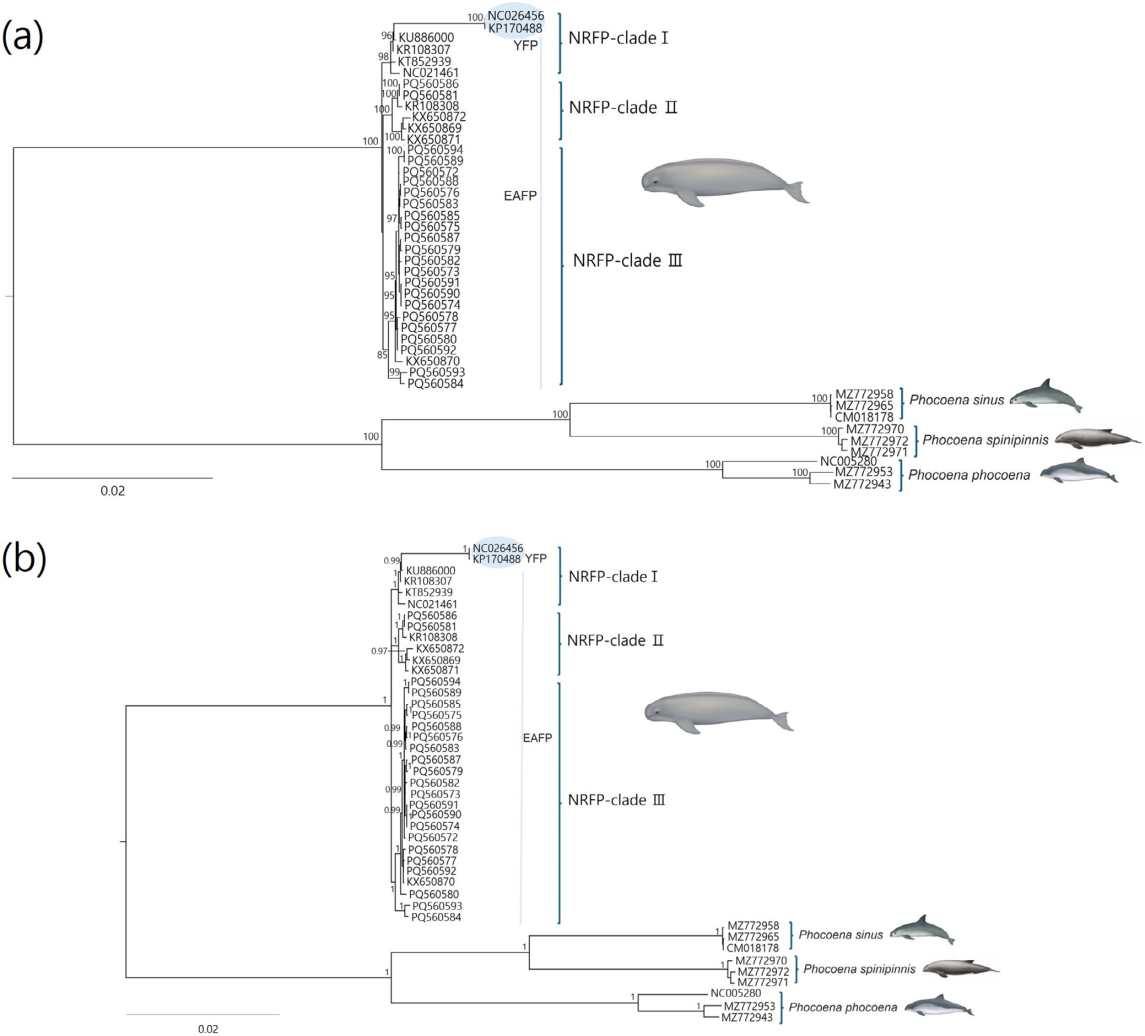
Phylogenetic trees inferred from the complete mitogenome of the narrow‐ridged finless porpoise, *Neophocaena asiaeorientalis* (16,413 bp). Three *Phocoena* species were used as outgroups. Both trees display three distinct clades with strong support values. (a) The maximum likelihood (ML) tree was constructed using the GTR + G substitution model, with bootstrap values indicated on branches (≥ 95%). (b) The Bayesian inference (BI) tree was constructed based on the HYK + I + G substitution model, with posterior probability values shown on branches (≥ 0.95).

**FIGURE 3:**
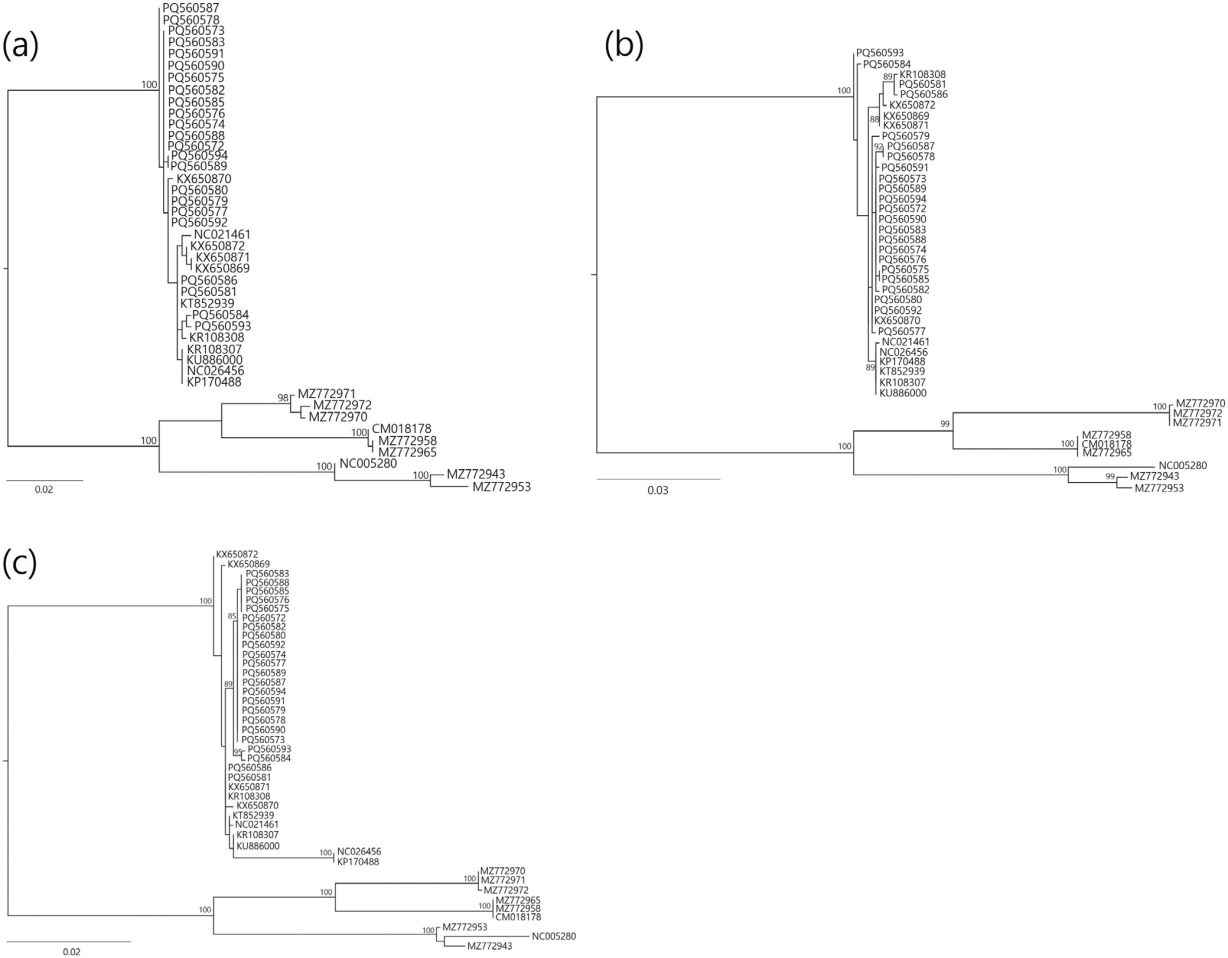
Phylogenetic trees inferred from partial mitochondrial genomes of the narrow‐ridged finless porpoise, *Neophocaena asiaeorientalis* , using the maximum likelihood (ML) method based on the GTR + G substitution model. Bootstrap values (≥ 85%) are indicated on branches. Three species from the genus *Phocoena* were used as outgroups. None of the partial gene regions fully resolved the three clades identified in the complete mitogenome analysis. (a) Phylogenetic tree based on the mitochondrial control region (CR, also known as the D‐loop, 928 bp). (b) Phylogenetic tree based on mitochondrial cytochrome b (*cyt b*, 1140 bp). (c) Phylogenetic tree based on mitochondrial cytochrome c oxidase 1 (*cox1*, 1555 bp).

The earliest divergence of finless porpoises within the family Phocoenidae was confirmed through both partial and complete mitogenome phylogenies (Figures 2 and 3), consistent with the findings of Chehida et al. (2020). ML and BI analyses of the complete mitogenome revealed three distinct monophyletic clades within *N. asiaeorientalis* (Figure 2a,b), each with strong support values (> 95% bootstrap in ML and > 95% posterior probability in BI). Six sequences from three subspecies clustered together in the first clade (GenBank accession numbers: NC026456, KP170488, and KU886000 for *N. a. asiaeorientalis*; KR108307 and KT852939 for *N. a. sunameri*; and NC021461 for *N. phocaenoides* ), with two sequences (NC026456 and KP170488) showing a high degree of divergence. The second clade consisted of two samples from this study and four previously published sequences from two subspecies (KR108308 for *N. a. asiaeorientalis* and KX650869, KX650871, and KX650872 for *N. a. sunameri*). The final clade included 21 sequences generated in this study, along with one earlier sequence (KX650870, listed as *N. phocaenoides* ). These findings show that the three subspecies of the genus *Neophocaena* were interspersed among the three clades. The sequences from the Southern Sea and the Korean Yellow Sea are mixed within the three clades, indicating no population structure based on sea region. Furthermore, phylogenetic trees based on partial sequences displayed different patterns compared to the complete mitogenome analyses, exhibiting little to no discrimination between sequences (Figure 3). All haplotypes of the complete mitogenome generated in this study have been deposited in the NCBI GenBank (16, 386 bp, accession numbers: PQ560572–PQ560594).

### 3.2 Genetic Diversity

Analysis of the complete mitogenome sequences from 23 individuals of the Korean population revealed that two individuals shared the same haplotype, resulting in 22 unique haplotypes. Nucleotide diversity was estimated at 0.142% (± 0.032%), while haplotype diversity was 99.6% (± 1.4%). A total of 134 polymorphic sites were identified, including 40 singletons and 94 parsimony‐informative sites. No insertion–deletion polymorphisms were detected among the 23 sequences (Table 4).

**TABLE 4:** Genetic diversity values of the complete mitogenome of the narrow‐ridged finless porpoise *(Neophocaena asiaeorientalis)* from the Korean sea.

Species	Location	*n*	Nh	Hd	π	P	S	PI
*Neophocaena asiaeorientalis sunameri*	South Korea	23	22	99.6% (± 1.4%)	0.142% (± 0.032%)	134	40	94

Abbreviations: Hd, haplotype diversity (± SD); *n*, number of examined sequences; Nh, number of haplotypes; P, polymorphic site; PI, parsimony informative sites; S, singleton; π, nucleotide diversity (± SD).

## 4 Discussion

The taxonomic classification of the finless porpoise has evolved with advances in molecular analysis techniques. Initially, *N. asiaeorientalis* was considered a subspecies of *N. phocaenoides* , but it has been given full species status following morphological and molecular studies in 2011 (Wang et al. 2008; Jefferson and Wang 2011). The two species can be distinguished by their dorsal ridges: *N. phocaenoides* has a broad, low ridge, while *N. asiaeorientalis* features a narrower, relatively higher ridge (Amano 2018). *Neophocaena phocaenoides* is distributed throughout the Indo‐Pacific, from the Persian Gulf to the Taiwan Strait and south to Indonesia. In contrast, *N. asiaeorientalis* is found exclusively in East Asian waters (Amano 2018). Consequently, *N. phocaenoides* is commonly known as the “Indo‐Pacific” finless porpoise, reflecting its broader distribution, while *N. asiaeorientalis* is referred to as the “narrow‐ridged” finless porpoise.

Both subspecies of *N. asiaeorientalis* —*N. a. sunameri* and *N. a. asiaeorientalis*—are facing severe conservation challenges due to rapidly declining populations. The IUCN Red List classifies *N. a. sunameri* as endangered and *N. a. asiaeorientalis* as critically endangered (Wang and Reeves 2017). As a result, conservation efforts in the East Asian regions, in which these subspecies are found, have become increasingly important, with research on these populations expanding in recent years. Population assessments have consistently reported significant declines in nearly all regions inhabited by the narrow‐ridged finless porpoise (Kasuya et al. 2002; Zhao et al. 2008; Shirakihara and Shirakihara 2013; Mei et al. 2014; Park et al. 2015; Hashimoto et al. 2015; Li et al. 2023). Therefore, it is critical to assess the extinction risk of this species through comprehensive evaluations and implement appropriate conservation strategies.

Additionally, increasing genetic and ecological evidence suggests that the Yangtze finless porpoise (*N. a. asiaeorientalis*) may warrant classification as a distinct species owing to its reproductive isolation from marine populations (Zhou et al. 2018). Thus, re‐examining the taxonomic distinctions using specimens from diverse regions is essential in developing more effective conservation strategies.

The six sequences in the first phylogenetic clade are all presumed to belong to *N. asiaeorientalis* (Figure 2). Of these, two sequences (KR108307 and KT852939) were explicitly identified as *N. a. sunameri*. Another sequence (NC021461), currently listed as *N. phocaenoides* and collected from Zhejiang Province in the East China Sea prior to the 2011 taxonomic revision, should likely be reclassified as *N. a. sunameri*. Similarly, a sequence from the Yangtze River, registered as *N. a. asiaeorientalis* (KU886000), appears more closely related to *N. a. sunameri* based on the phylogenetic analysis. The remaining two sequences (KP170488 and NC026456), both from the Yangtze River, were confirmed as *N. a. asiaeorientalis*. Several sequences in this clade lack verification by published sources, and the collection locations are uncertain, requiring further investigation. Based on available data, we classify only KP170488 and NC026456 as *N. a. asiaeorientalis*, while the other four sequences are identified as *N. a. sunameri*. This suggests significant genetic divergence between the Yangtze River population (*N. a. asiaeorientalis*) and the East Asian population (*N. a. sunameri*), which aligns with the findings of Zhou et al. (2018).

The specimen linked to KR108308 in the second clade, collected from the Northern Yellow Sea, was likely misidentified as *N*. *a. asiaeorientalis* and is likely *N. a. sunameri*. For the remaining three sequences currently registered as *N. a. sunameri*, the collection locations need to be confirmed. The final clade included one previously published sequence (KX650870) alongside sequences generated in this study. Although listed as *N. phocaenoides* , the fact that the collection site for this specimen remains unknown suggests that it may have been misidentified and could instead belong to *N. a. sunameri*, consistent with other sequences in the same clade. The inferred species classification for all finless porpoise sequences used in the phylogenies is summarized in Table 3.

The two partial mitochondrial genes commonly used in cetacean phylogenetic studies, mtDNA CR and *cyt b*, were insufficient to delineate the three clades identified by complete mitogenome analysis. They also failed to capture the significant divergence of the two Yangtze finless porpoise sequences (Figure 3a,b). In contrast, while the *cox1* gene—rarely used in cetacean phylogenies—did not clearly resolve the three clades, it did account for the substantial divergence of the Yangtze finless porpoise sequences (Figure 3c). These findings highlight the importance of using complete mitogenome sequences to accurately reconstruct the evolutionary history of closely related taxa. Additionally, a previous study found that among the Korean, Chinese, and Japanese populations, only the Japanese population is genetically isolated (Lee et al. 2019). Morphological differences, such as dorsal ridge shape and total body length, have also been observed between the Korean and Japanese populations, even within the same subspecies (personal communication). Therefore, comprehensive phylogenetic analyses that include all populations of the species are needed. The tiling multiplex PCR and NGS approach developed in this study can efficiently generate complete mitogenomes, improving our understanding of the phylogenetic relationships among these populations.

Assessing genetic diversity is vital for developing effective conservation strategies, as its preservation is a cornerstone of conservation biology. A previous study identified a significant correlation between genetic diversity and a species' IUCN Red List status (Chehida et al., 2020). Low genetic diversity can diminish a species' ability to adapt to environmental changes and resist disease, increasing the risk of extinction (Lacy 1997; Frankham 1999, 2002). In this context, the haplotype and nucleotide diversities of the Korean population in this study were similar to the averages reported in previous research (Chehida et al., 2020). Differences may be attributed to variations in sample size or potential misidentifications in the previous study, as discussed in the phylogenetic analysis.

Smaller populations generally exhibit reduced genetic diversity (Amos and Balmford 2001), a trend also observed in narrow‐ridged finless porpoises (Lee et al. 2019). In East Asian waters, these porpoises show both low genetic diversity and high genetic differentiation when compared with the genetic diversity of other phocoenid species, such as *Phocoena phocoena* and *Phocoenoides dalli* , indicating limited gene flow between populations (Lee et al. 2019). Reduced gene flow increases vulnerability to environmental changes and human activities, limiting a population's ability to adapt through genetic variation. The Korean population, which has the highest genetic diversity and lowest genetic differentiation, plays a critical role in preserving the species' overall genetic diversity (Lee et al. 2019). This highlights the importance of the Korean population in maintaining the species' genetic health and long‐term survival. However, this population has experienced a sharp decline, primarily owing to anthropogenic threats such as entanglement in fishing nets, boat strikes, and habitat loss, as these porpoises inhabit coastal areas. The population in the Korean Yellow Sea decreased from 36,000 individuals in 2004/2005 to 13,000 individuals in 2011, a decline of approximately 70% over 7 years (Park et al. 2015). Without targeted conservation efforts, both population size and genetic diversity are expected to decline further. Therefore, ongoing monitoring of genetic diversity, in conjunction with population assessments, is crucial. Given the essential role of the Korean population in conserving the species, both national and international cooperation are needed to develop and implement effective conservation strategies.

## Author Contributions

**Sunmin Kim:** conceptualization (supporting), data curation (equal), formal analysis (equal), investigation (equal), methodology (equal), project administration (equal), resources (equal), software (equal), validation (equal), writing – original draft (lead). **Youngran Lee:** funding acquisition (equal), methodology (equal), project administration (equal), resources (equal). **Dong‐Yeop Lee:** formal analysis (equal), investigation (equal), methodology (equal), software (equal), visualization (equal), writing – original draft (supporting). **Heesu Lee:** investigation (equal), methodology (equal). **Seon‐Mi Lee:** writing – review and editing (supporting). **Nari Kim:** resources (supporting), writing – review and editing (supporting). **Seongjun Choe:** writing – review and editing (supporting). **Dong‐Hun Lee:** conceptualization (lead), data curation (equal), formal analysis (equal), funding acquisition (equal), investigation (equal), methodology (equal), project administration (equal), resources (equal), software (equal), supervision (lead), validation (lead), visualization (equal), writing – review and editing (lead).

## Conflicts of Interest

The authors declare no conflicts of interest.

## Data Availability

The genome sequence data presented in this study has been deposited in the NCBI GenBank under the accession number PQ560572–PQ560594.
