# Correction to “Complete Mitochondrial Genome Analysis Reveals Genetic Diversity in the Narrow–Ridged Finless Porpoise ( *Neophocaena asiaeorientalis* ) Across East Asian Waters”

**DOI:** 10.1002/ece3.71436

**Published:** 2025-05-13

Kim, S., Lee, Y., Lee, D.‐Y., Lee, H., Lee, S.‐M., Kim, N., Choe, S., and Lee, D.‐H. (2025), Complete Mitochondrial Genome Analysis Reveals Genetic Diversity in the Narrow–Ridged Finless Porpoise ( *Neophocaena asiaeorientalis* ) Across East Asian Waters. *Ecology and Evolution*, 15: e71229. https://doi.org/10.1002/ece3.71229.

In the published article, the following errors were identified:

1. In the Introduction, the authority “*N. a. asiaeorientalis* (Yangtze finless porpoise, Rudolph and Smeenk 2009)” is incorrect. This should be as follows: “*N. a. asiaeorientalis* (Yangtze finless porpoise, Pilleri and Gihr 1972).”
2. Throughout the article, the citation “Chehida et al. (2020)” is incorrect. This should be as “Ben Chehida et al. (2020).”
3. In Table 3, categories *Neophocaena* and *Phocoena* (outgroup) were incorrectly shaded yellow, and the citation “Arnason et al. (2004)” contains a spelling error. This should be as “Árnason et al. (2004).”
4. In the References section, the authors' names in the following reference are incorrect “Árnason, U., A. N. E. T. T. E. Gullberg, and B. E. N. G. T. Widegren. 1993. ‘Cetacean Mitochondrial DNA Control Region: Sequences of all Extant Baleen Whales and Two Sperm Whale Species’ *Molecular Biology and Evolution* 10, no. 5: 960–970. https://doi.org/10.1093/oxfordjournals.molbev.a040061.” The correct reference is “Árnason, U., A. Gullberg, and B. Widegren. 1993. ‘Cetacean Mitochondrial DNA Control Region: Sequences of all Extant Baleen Whales and Two Sperm Whale Species’ *Molecular Biology and Evolution* 10, no. 5: 960–970. https://doi.org/10.1093/oxfordjournals.molbev.a040061.”

The online version of this article has been corrected accordingly.

We apologize for these errors.

